# Skin-analogue primary poroid neoplasms of the head and neck with *YAP1/WWTR1::MAML2/NUTM1* fusions: clinicopathologic and genetic spectrum of a novel tumor family delineated in a series of 10 cases

**DOI:** 10.1007/s00428-025-04369-y

**Published:** 2025-12-06

**Authors:** Abbas Agaimy, Elan Hahn, Juan C. Hernandez-Prera, Diana Bell, Yen Chen Kevin Ko, Justin Bubola, Deepika Chugh, Thomas Cramer, Sarina K. Mueller, Lars Tögel, Robert Stoehr, Ilan Weinreb, Justin A. Bishop

**Affiliations:** 1 Institute of Pathology, Erlangen University Hospital, Friedrich Alexander University of Erlangen-Nuremberg, Krankenhausstrasse 8-10, 91054 Erlangen, Germany; 2 Comprehensive Cancer Center European Metropolitan Area Erlangen-Nuremberg (CCC ER-EMN), Erlangen, Germany; 3 Department of Pathology and Laboratory Medicine, Mount Sinai Hospital, Toronto, ON Canada; 4 Department of Pathology, Moffitt Cancer Center, Tampa, FL USA; 5 Head and Neck/Endocrine Pathology Center of Excellence, Division of Anatomic Pathology, University of Pittsburgh Medical Center, Pittsburgh, PA USA; 6 Department of Pathology and Laboratory Medicine, University of British Columbia, Vancouver, Canada; 7 Department of Oral Biological and Medical Sciences, Faculty of Dentistry, University of British Columbia, Vancouver, Canada; 8 Oral Pathology and Oral Medicine, Faculty of Dentistry, University of Toronto, Toronto, ON Canada; 9 Department of Otorhinolaryngology, Head and Neck Surgery, Bundeswehrkrankenhaus Berlin, Berlin, Germany; 10 Department of Otorhinolaryngology, Head and Neck Surgery, Erlangen University Hospital, Friedrich Alexander University of Erlangen-Nuremberg, Erlangen, Germany; 11 Laboratory Medicine Program, University Health Network, Toronto General Hospital, Toronto, ON Canada; 12 Department of Pathology, University of Texas Southwestern Medical Center, Dallas, TX USA; 13 Department of Laboratory Medicine and Pathobiology, University of Toronto, Toronto, ON Canada

**Keywords:** Eccrine poroma, Squamous cell carcinoma, Bromodomain inhibitors, Molecular profiling, Targeted therapy

## Abstract

Porocarcinoma is a rare type of aggressive skin adnexal carcinoma with terminal sweat duct differentiation, representing the malignant counterpart of the more common benign eccrine poroma. Diagnosis is based on distinctive morphology, supplemented by immunohistochemistry. While all tumors display a squamous immunophenotype, porocarcinomas only infrequently show frankly squamous cell cytology (squamous variant). Most poromas and porocarcinomas harbor *YAP1::MAML2/NUTM1* or, rarely, *WWTR1::NUTM1* fusions. We herein describe 10 extra-cutaneous primary (8 salivary, 1 mandibular, 1 nasopharyngeal) poroid neoplasms affecting 6 males and 4 females, aged 17 to 71 years (median, 49). The salivary cases originated in the parotid (*n* = 6; 1 within intraparotid lymph node), lower lip minor salivary glands (1), and palate (1). All, but one case, were treated by excision with or without lymph node dissection (two received adjuvant chemoradiation). Immune chemotherapy was given to the nasopharyngeal case. Histologically, five cases were frankly malignant (porocarcinoma-like), two infiltrating low-grade and three bland-looking (poroma-like). Three bland tumors were predominantly cystic. Regional lymph nodes were positive in three of three sampled cases. Limited follow-up was available for six patients (1–43 months; median, 6). Five patients were disease-free and one was alive with disease at 16 months. Targeted RNA sequencing revealed *YAP1* fusions in nine cases (fused to *MAML2* in four and to *NUTM1* in five cases) and a *WWTR1::NUTM1* fusion in one case. This series introduces skin-analogue poroid neoplasms as a distinct extra-cutaneous head and neck entity, originating mostly from major and minor salivary glands and recapitulating/spanning the spectrum of cutaneous poroid tumors with some cases showing bland poroma-like morphology, while others are high-grade porocarcinoma-like malignancies. This tumor type should be distinguished from the many primary and metastatic salivary neoplasms with squamous immunophenotype. In particular, the *NUTM1*-rearranged (NUTM1-immunopositive) cases should be distinguished from the more aggressive NUT carcinoma, as both share overlapping anatomic sites and squamous cell phenotype and express NUTM1. Moreover, *MAML2*-fused cases might be confused with variant or high-grade mucoepidermoid carcinomas, as they share squamous phenotype and *MAML2* rearrangements. The biology of bland cases and their nosology (benign vs low-grade malignant) remain to be further characterized.

## Introduction

Salivary gland carcinomas with squamous cell immunophenotype have been a subject of controversy due to the notion that nearly all squamous cell carcinomas (SCC) of the major salivary glands (mostly the parotid) represent metastases from cutaneous origin [1]. However, over the years and with increasing molecular-informed refinement of tumor classification, it became evident that the mere presence of squamous immunophenotype does not equal SCC in a broad nosological sense. Specifically, lymphoepithelial carcinoma (EBV-associated in most cases [2]) and hyalinizing clear cell carcinoma (*EWSR1* fusion-associated [3]) are two other entities with distinctive clinicopathological profiles but showing squamous cell immunophenotype. Moreover, adamantinoma-like Ewing sarcoma has been redefined as a distinctive *EWSR1/FUS::ETS* fusion sarcoma with squamous immunophenotype, frequently expressing low and high molecular weight keratins and p63/p40 in addition to other markers [4–6]. In addition, NUT carcinoma has been delineated as a rare but well documented, molecularly defined salivary gland carcinoma with squamous cell immunophenotype in majority of cases [7]. Finally, salivary gland carcinomas showing SCC phenotype and displaying non-descript transitional cell-like morphology have been recently shown to harbor recurrent *FGFR3::TACC3* fusions in favor of a putative entity [8].

We herein report detailed clinicopathological and molecular findings of 10 head and neck neoplasms showing morphological, immunophenotypic, and molecular features of eccrine poroma/porocarcinoma of the skin [9, 10], but originating in non-cutaneous sites (major and minor salivary glands, nasopharynx, mandible) in the absence of skin primary. Our study establishes “skin-analogue poroid neoplasms” as a distinctive novel salivary entity and suggests underecognition of this nearly unreported tumor type at these sites.

## Materials and methods

The cases have been retrieved from our routine and consultation files. Three cases have been reported previously under different names (cases 1, 4, and 6 in Table 1 [11–13]) but extended follow-up was obtained and critical histological reappraisal of the entity was undertaken. All available H&E-stained slides have been critically re-reviewed. Due to the consultation nature of most cases, immunohistochemistry (IHC) was performed in different laboratories and the stains applied varied from case to case, based on tissue availability and initial differential diagnostic considerations (details of the staining protocols and antibody sources are available upon request).

**Table 1:** Clinicopathological features of skin-analogue poroid neoplasms of the head and neck (*n* = 10)

No	Age/sex	Site/size cm	Symptoms	Treatment	Nodal status	Outcome	Fusion
1 [11]	24/M	Parotid/2	Growing mass since 6 mo, B-symptoms (Fever, weight loss, night sweat)	Total parotidectomy, neck dissection, aRCT	+ (3/16)	NED (43 mo)	*YAP1::MAML2*
2	62/M	Parotid/3.8	Mass	Total parotidectomy, neck dissection	+	Recent case	*YAP1::MAML2*
3	67/F	Parotid LN/0.6	Parotid nodule	Total parotidectomy, neck dissection	Neg*	NED (7 mo)	*WWTR1::NUTM1*
4 [12]	17/M	Lower lip/NA	Painless swelling, old mucocele?	NA	NA	NA	*YAP1::NUTM1*
5	71/F	Nasopharynx/fossa of Rosenmueller/2.2	Prior history of NPC (12 years before) CRT, with CR (not clear if recurrence or new malignancy)	CT (Taxol/fluorouracil/hydroxyurea) + re-RT + pembrolizumab	Not sampled	Progression/residual disease (16 mo)	*YAP1::MAML2*
6 [13]	37/M	Parotid/3.8	Parotid mass and associated adenopathy	Total parotidectomy, neck dissection	+ (pN3b)	Positive neck LNs, no follow up	*YAP1::MAML2*
7	70/F	Parotid/1.3	Growing mass for 4 mo NED of post-Op CT Neck and PET	Excisional biopsy and adjuvant radiotherapy	Not sampled	NED (5 mo)	*YAP1::NUTM1* Low TMB, MSS, no UV mutation signature
8	27 M	Parotid/0.5	Incidentally discovered during imaging studies for sinusitis	Excision	Not sampled	Recent consult case	*YAP1::NUTM1*
9	24/F	Mandible/NA	Odontogenic lesion	Curettage	NA	NED (1 mo)	*YAP1::NUTM1*
10	69/M	Palate/NA	Mass	Excision	NA	NED (1 mo)	*YAP1::NUTM1*

*aRCT* adjuvant radiochemotherapy, *CT* chemotherapy, *LN* lymph node, *mo* month, *MSS* microsatellite stable, *NA* not available, *NED* no evidence of disease, *NPC* nasopharyngeal carcinoma, *RT* radiotherapy, *UV* ultraviolet, *TMB* tumor mutation burden

*Tumor limited to an intraparotid node, no other primary

### Next-generation sequencing

For cases 1, 2, and 3, RNA was isolated from formalin-fixed paraffin-embedded (FFPE) tissue sections using RNeasy FFPE Kit of Qiagen (Hilden, Germany) and quantified spectrophotometrically using NanoDrop 1000 (Waltham, USA). Molecular analysis was performed using the TruSight RNA Fusion panel (Illumina, Inc., San Diego, CA, USA) with 500 ng RNA as input according to the manufacturer’s protocol. Libraries were sequenced on a MiSeq (Illumina, Inc., San Diego, CA, USA) with > 3 million reads per case, and sequences were analyzed using the RNA-Seq alignment workflow version 2.0.1 (Illumina, Inc., San Diego, CA, USA) and the Arriba fusion detection algorithm version 2.5.0 [14]. Potential gene fusion events were confirmed by visualization of clusters of soft-clipped sequences at breakpoint sites in the Integrative Genomics Viewer (IGV) version 2.2.13 (Broad Institute). Cases 4, 5, and 6 were analyzed for gene fusions using the methods reported previously [12, 13, 15]. Cases 7 and 8 were analyzed by Archer FusionPlex Pan Solid Tumor v2 Panel (Integrated DNA Technologies, Inc. Boulder, CO, USA). For cases 9 and 10, RNA was extracted using RNeasy FFPE Kit of Qiagen (Hilden, Germany) and the quality of the extracted RNA was assessed using the Agilent 2100 Bioanalyzer and the RNA 6000 Nano kit to ensure that it met the input requirement of > 8 ng/mL. The cases were sequenced using the Illumina NovaSeq 6000 (Illumina, San Diego, CA, USA) platform following enrichment with the Illumina RNA Prep with Enrichment cDNA synthesis kit (Illumina, San Diego, CA, USA) and a custom probe set (Twist Bioscience, South San Francisco, CA, USA). Somatic variants and gene fusions were identified using the DRAGEN Bio-IT Platform (v4.0.3) (Illumina, San Diego, CA, USA) aligned to the GRCh37/hg19 reference genome and annotated with public and internal databases.

## Results

### Clinical findings (Table 1)

Patients were 6 males and 4 females aged 17 to 71 years (median, 49). Eight tumors originated in the salivary glands: six in the parotid gland (one within an intraparotid lymph node), and one each in the minor salivary glands of lower lip and palate. Non-salivary cases originated in the nasopharynx (*n* = 1) and mandible (*n* = 1). Their size ranged from 0.5 to 3.8 cm (median, 2). Surgical excision with/without lymph node dissection was treatment of all but one case (two received adjuvant radiochemotherapy). Immune chemotherapy was given to the nasopharyngeal tumor. Regional nodes were positive in all 3 sampled cases. Limited follow-up was available for 6 patients (1–43 months; median, 6). Five patients were disease-free, and one was alive with disease at 16 months.

### Pathological findings in high-grade and low-grade poroid carcinomas (*n* = 7)

**Fig. 1:**
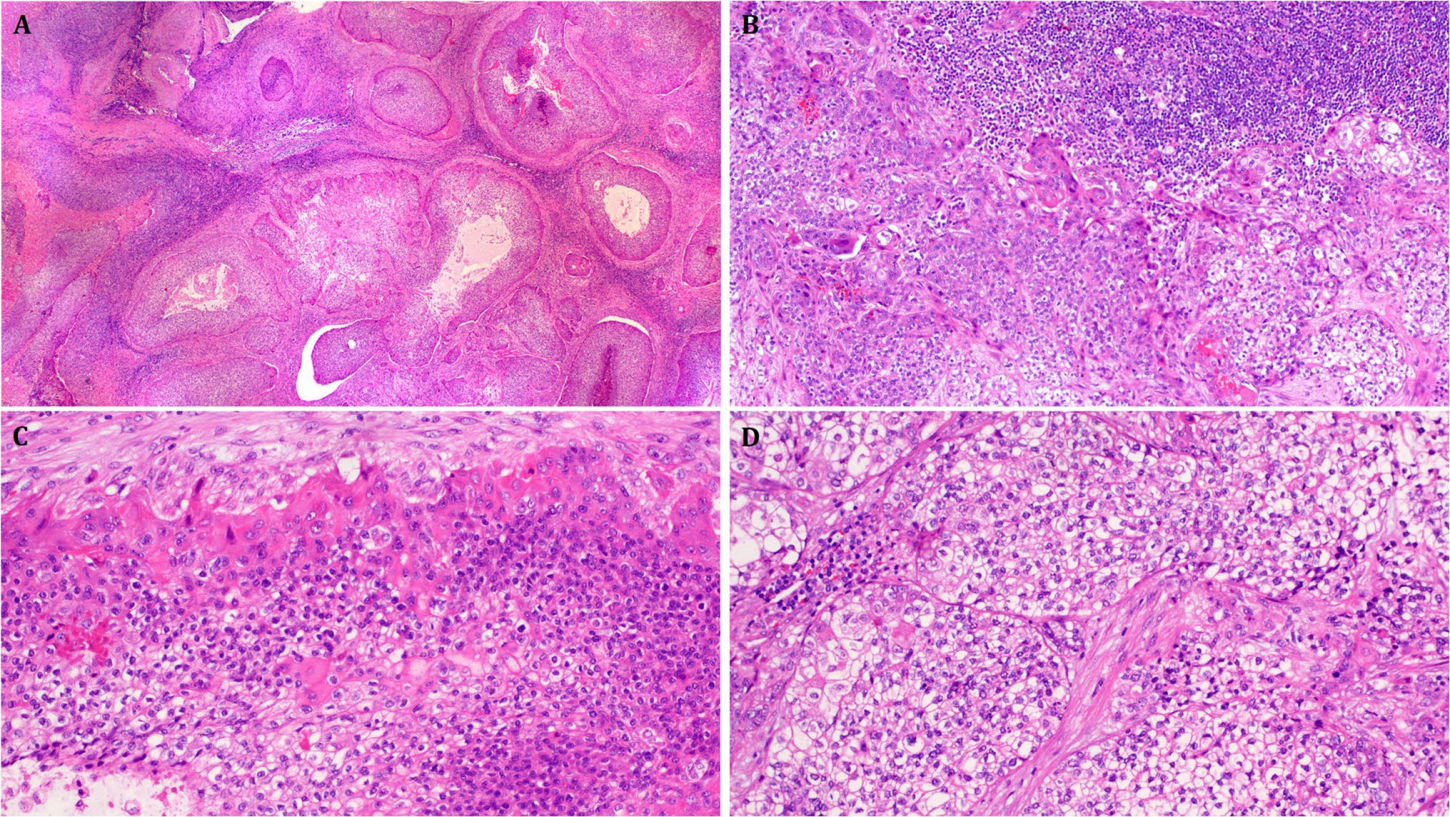
High-grade poroid carcinoma of parotid (case #2) showing multiple large lobules with prominent central comedo-type necrosis (**A**). **B** variable squamoid eosinophilic (left) and clear cells (right) are seen surrounded by prominent lymphoid cuff. **C** Zonation with eosinophilic cutical cells at periphery surrounding basophilic poroid cells (right) and clear cells (lower left). **D** Higher magnification of the clear cell areas

**Fig. 2:**
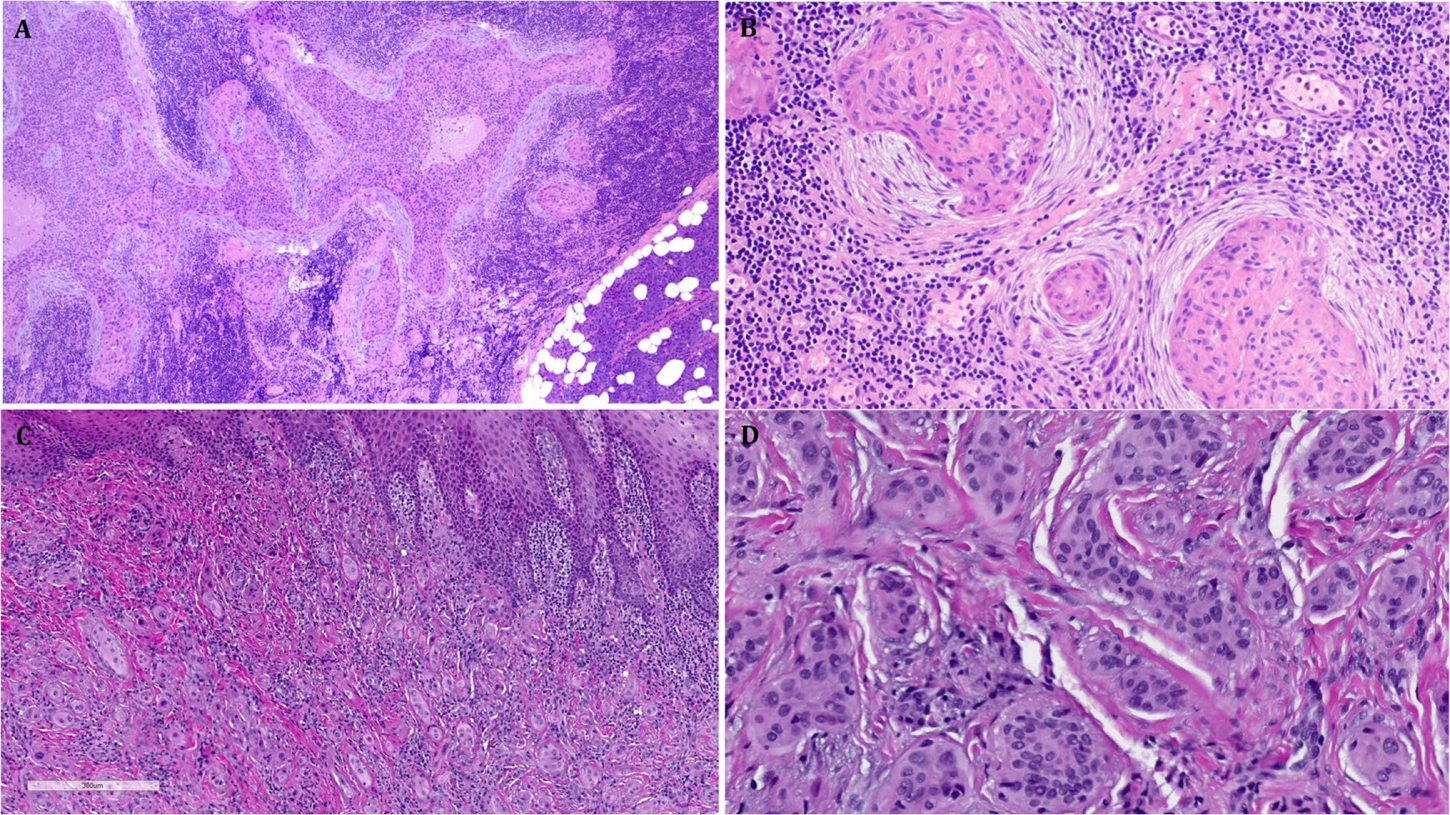
**A + B** This squamoid poroid carcinoma (case #3) was limited to an intraparotid node, note prominent small nesting pattern surrounded by concentric desmoplasia. **C + D** This diffusely infiltrative poroid carcinoma originated from minor salivary glands of the lip (case #4) showed prominent epitheliotropism (**C**) and small nesting, mimicking a syringoid neoplasm (**D**)

**Fig. 3:**
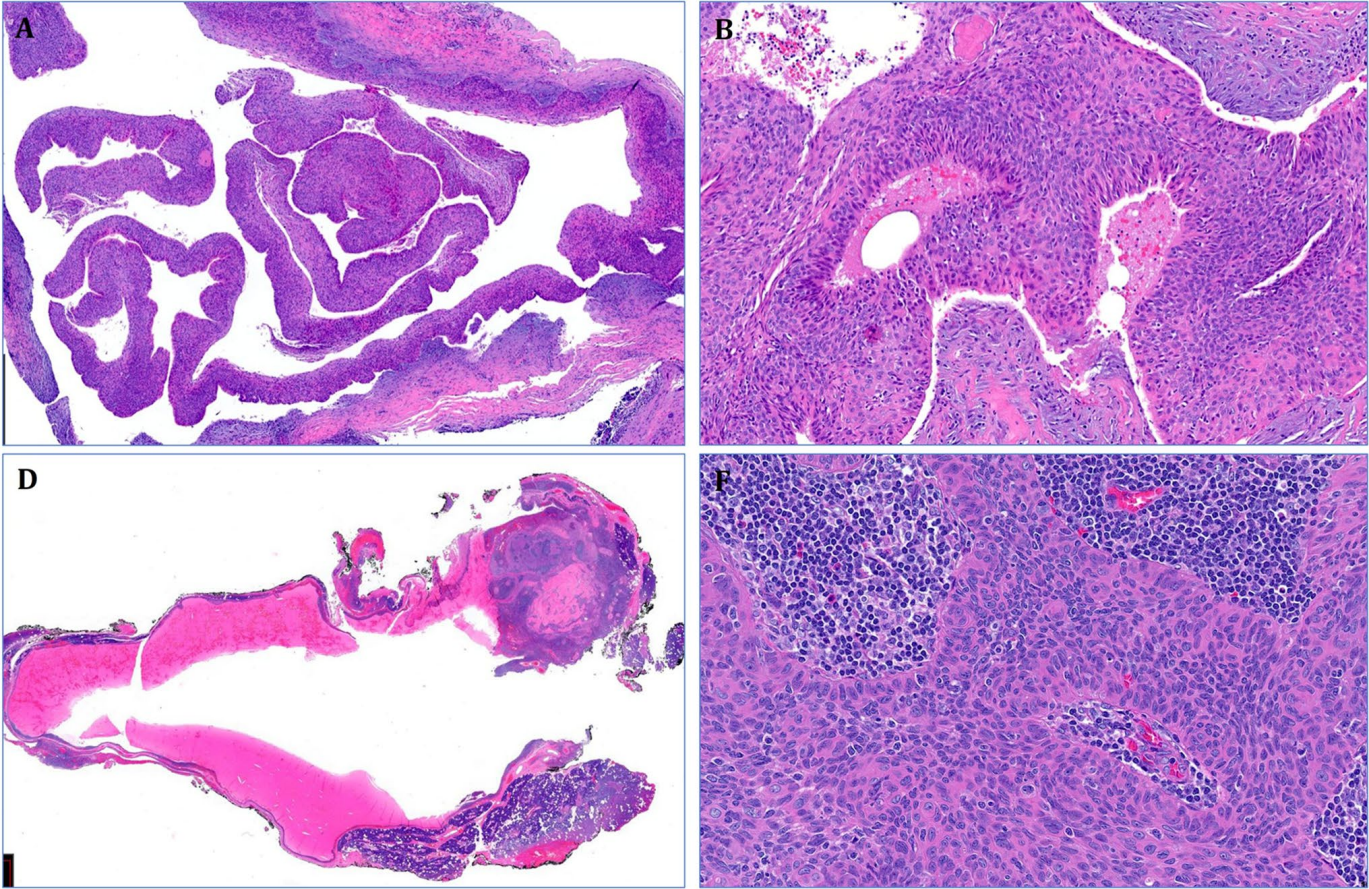
Case #7 (**A**, **B**) and case #8 (**C**,** D**) were predominantly cystic, composed of monomorphic low-grade basophilic poroid cells with variable infiltrating component surrounded by either desmoplastic reaction (**B**) or lymphoid stroma (**D**)

Histologically, seven tumors were variably infiltrative and non-encapsulated with either high-grade frankly malignant cytology (cases 1–5) or displaying low-grade but infiltrating histology (cases 6 and 7), hence qualifying as malignant poroid neoplasms (analogous to cutaneous porocarcinomas). They were composed of monomorphic medium-sized round to oval cells with variable epidermoid and basaloid cytological appearance. They are disposed into irregularly branching, communicating, well-circumscribed variably sized compact aggregates and distinct lobules within a fibroinflammatory desmoplastic stroma. All tumors contained a variable lymphoid reaction that was most prominent at the tumor periphery, occasionally mimicking a lymph node. Cytologically, a variable combination of small basophilic poroid cells and slightly larger eosinophilic cuticular cells was seen in all cases, occasionally with zonal distribution with the cuticular cells being more prominent towards the periphery of the aggregates (Fig. 1). One tumor showed prominent clear cell component (Fig. 1). A distinctive small-nested pattern was seen in two tumors (Fig. 2). Two parotid tumors showed infiltrative growth of low-grade poroid cells with prominent cystic pattern (Fig. 3) and extensive lymphoid background stroma. Two tumors (cases 1 and 6) were predominantly squamous with abrupt pearls and contained amphophilic cells with connection to native ducts. One of the squamoid cases showed multiple follicle-like concentric aggregates. However, subtle minor squamous features/squamous cell aggregates were recognizable in almost all cases if thoroughly searched for. Mitotic activity was brisk in 5 high-grade cases. Two tumors showed prominent foci of central comedonecrosis.

### Pathological findings in bland-looking (poroma-like) poroid neoplasms (*n *= 3)

**Fig. 4:**
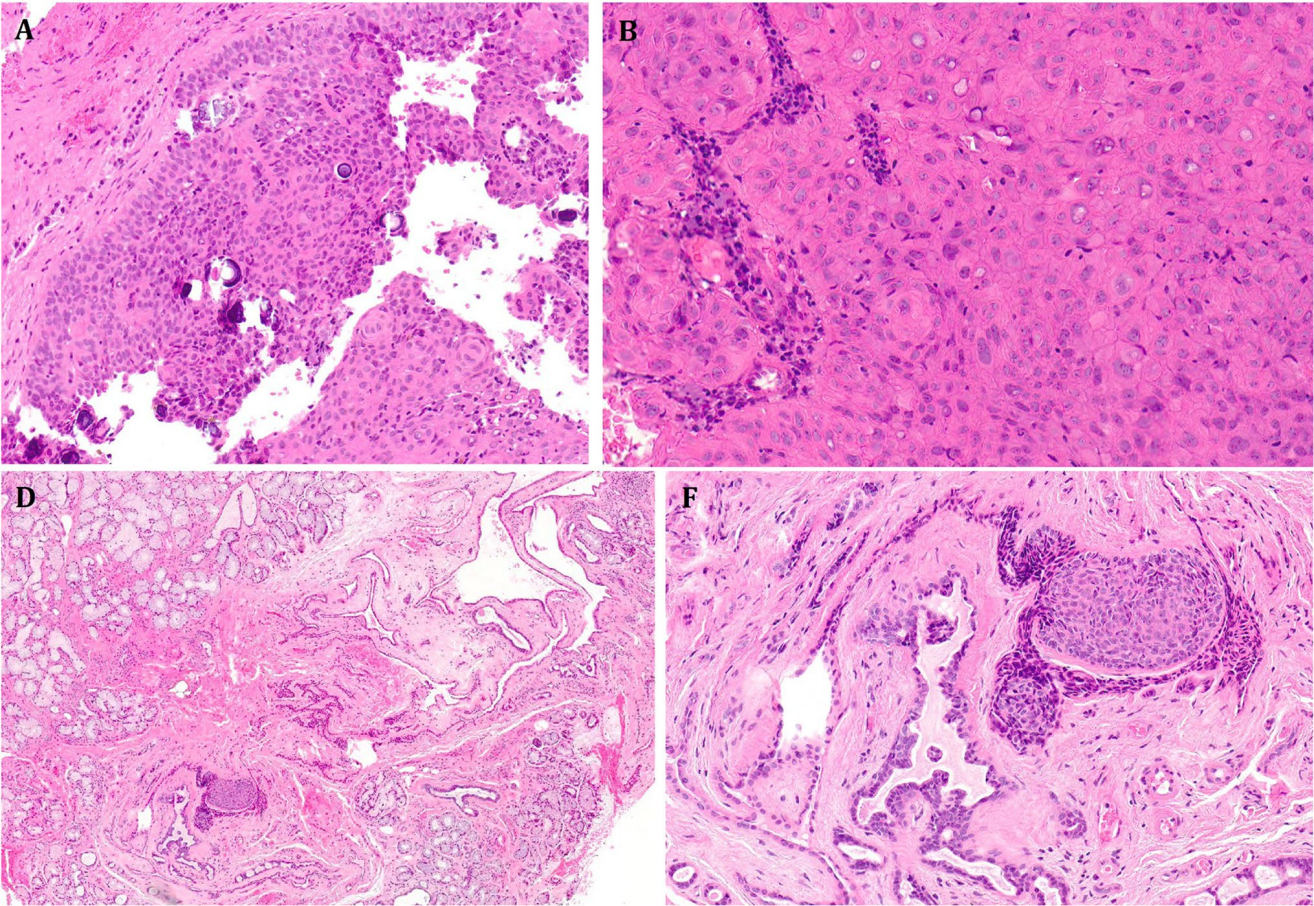
**A** +** B** Case #9 displayed bland-looking monomorphic eosinophilic cuticular-like cells, disposed into solid sheets with prominent psammoma bodies (in **A**). C + D Case #10 originated from the palatine minor salivary glands, closely associated with the duct system mimicking cystadenoma (dermal duct tumor-like). **D** Higher magnification showing bland monomorphic basophilic poroid cells

Three tumors (cases 8–10) were cytologically bland and variably cystic without clearly invasive growth. These have been originally diagnosed as cystadenoma (1), myoepithelioma (1), and odontogenic tumor, suspicious for carcinoma (1). The odontogenic tumor contained foci of psammomatoid calcifications and hyaline deposits (Fig. 4).

**Fig. 5:**
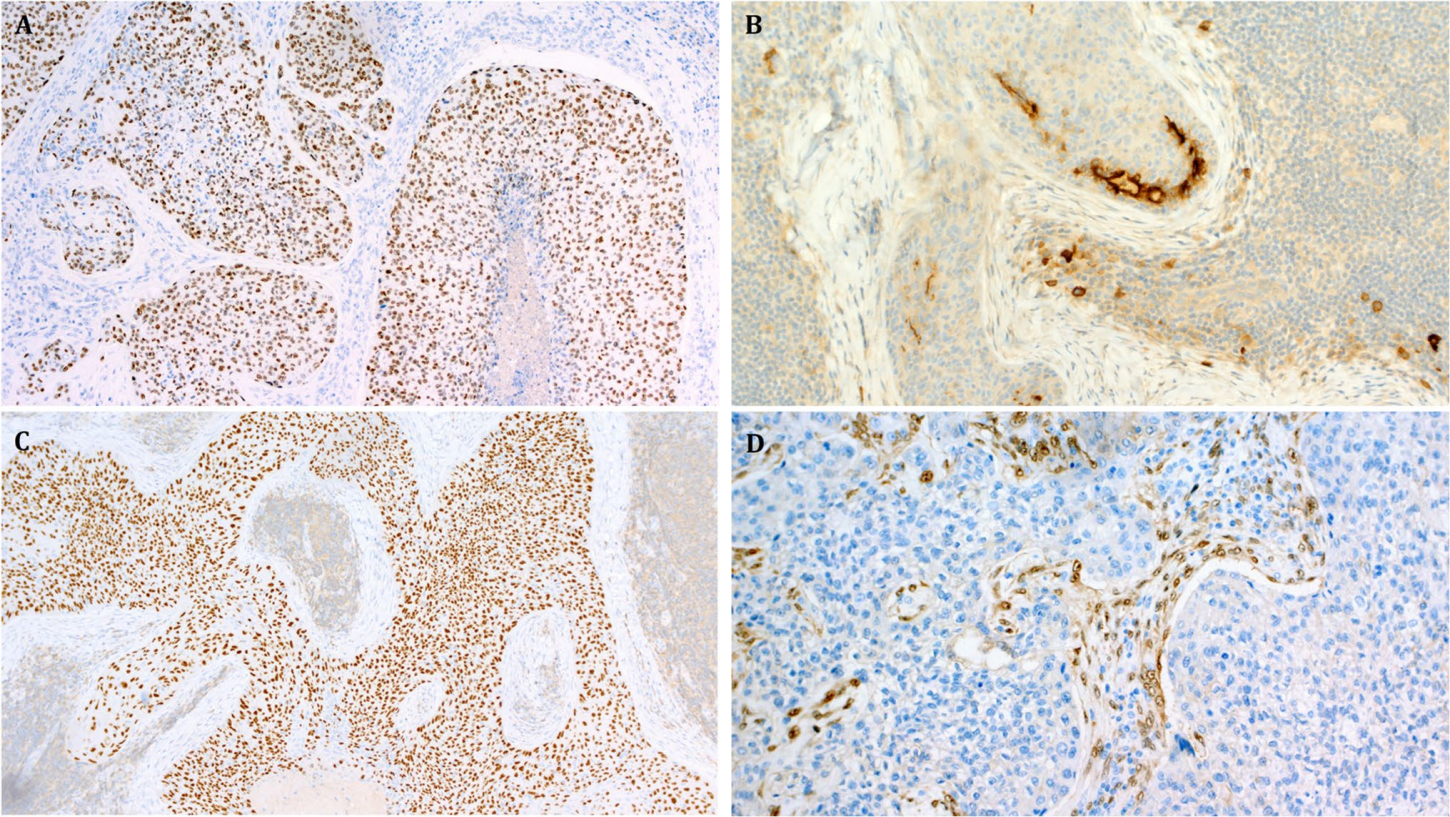
Representative immunohistochemical findings. **A** Diffuse strong expression of p40 (case #2). **B** EMA highlighting focal ductal component (case #3). **C** Strong NUT expression (case 3). **D** Loss of YAP1 C-terminus with preserved expression in the background stromal cells (case #2)

By immunohistochemistry, the tumors were consistently positive with p63 (6/6), p40 (4/4), either p63 or p40 (8/8; Fig. 5A), CK5/6 (4/4), EMA (2/2; Fig. 5B), and CK7 (3/3). NUT was positive in 5/5 *NUTM1*-rearranged tumors and negative in 2 of 2 tumors without *NUTM1* fusion (Fig. 5C). p16 was negative or only patchy positive in 4/5 tumors. One p16-positive tumor lacked high-risk HPV by RNA in situ hybridization. Other negative tested markers are included in Table 2. Two tumors with *YAP1* fusions have been tested with the C-terminus YAP1 antibody; both showed complete loss of expression (Fig. 5D). The *WWTR1::NUTM1*-fused case revealed retained YAP1 expression.

**Table 2:** Pathological and molecular features of skin-analogue poroid neoplasms of the head and neck (*n* = 10)

Not	Original/submitted diagnosis	Histological pattern	IHC +	IHC-	Genotype
1	Keratinizing Squamous cell carcinoma	Frankly malignant (porocarcinoma-like)	p63, CK5/6	AR, NUT, p16, p53 wildtype. HPV-(DNA-based)	***YAP1(ex5)::MAML2(ex2)***
2	Salivary duct carcinoma? Metastasis?	Frankly malignant (porocarcinoma-like)	p40, CK7, focal CD117	SOX10, S100, AR, SMA, p16, HER2, NUT, MUC4, ALK, Pan-TRK, NR4A3, CD5, p53 wildtype	***YAP1(ex1)::MAML2(ex2)***
3	Squamoid porocarcinoma	Frankly malignant (porocarcinoma-like)	p63, p40, NUT, EMA focal, P53	p16	***WWTR1(ex3)::NUTM1(ex3)***
4	Squamous cell carcinoma	Frankly malignant (porocarcinoma-like)	NUT, SOX-10, pankeratin, p40 (weak, focal), p63 (weak), focal & peripheral SMA and S100	PAX8, HMB45, wild type p53	***YAP1(ex2)::NUTM1(ex5)***
5	Nonkeratinizing (differentiated) carcinoma with squamoid features	Frankly malignant (porocarcinoma-like)	p40 - diffuse and strong, CK5/6 -diffuse and strong; PD-L1 22C3: CPS:15	p16 (40%, patchy reactivity); EBER, high-risk HPV-(RNAScope HPV HR18)	***YAP1(ex4)::MAML2(ex2)*** *BAP1* variant (c.983dupC; p.P329fs*69) (VAF: 41%), *TP53* (c.375G > A; p.T125T) (VAF: 44%)
6	Low-grade adenocarcinoma NOS	Infiltrating low-grade	None performed	None performed	***YAP1(ex1)::MAML2(ex2)***
7	Squamous cell carcinoma	Infiltrating low-grade	p63 and CK5/6 diffusely and strongly positive, p16 positive, NUT	High-Risk HPV (RNAScope HPV HR18)	***YAP1(ex5)::NUTM1(ex5)***
8	Myoepithelioma	Bland-looking (poroma-like)	p63, SOX10, CK7, CAM 5.2, CK5/6 and NUT, EMA variable +. Ki67 1–2%	S100, DOG1, SMA, PLAG1, NRASQ61R, and beta-catenin	***YAP1(ex5)::NUTM1(ex4)***
9	Atypical epithelial odontogenic tumor with CEOT-like features, suspicious for carcinoma	Bland-looking (poroma-like)	P53 overexpressed	-	***YAP1 (ex2)::NUTM1 (ex4) NM_001284292***
10	Cystadenoma	Bland-looking (poroma-like)	Bilayered, CK7 positive in both, S100, SOX10, p63 variable	Mammaglobin	***YAP1 (ex2)::NUTM1 (ex3)***

### Molecular findings

**Fig. 6:**
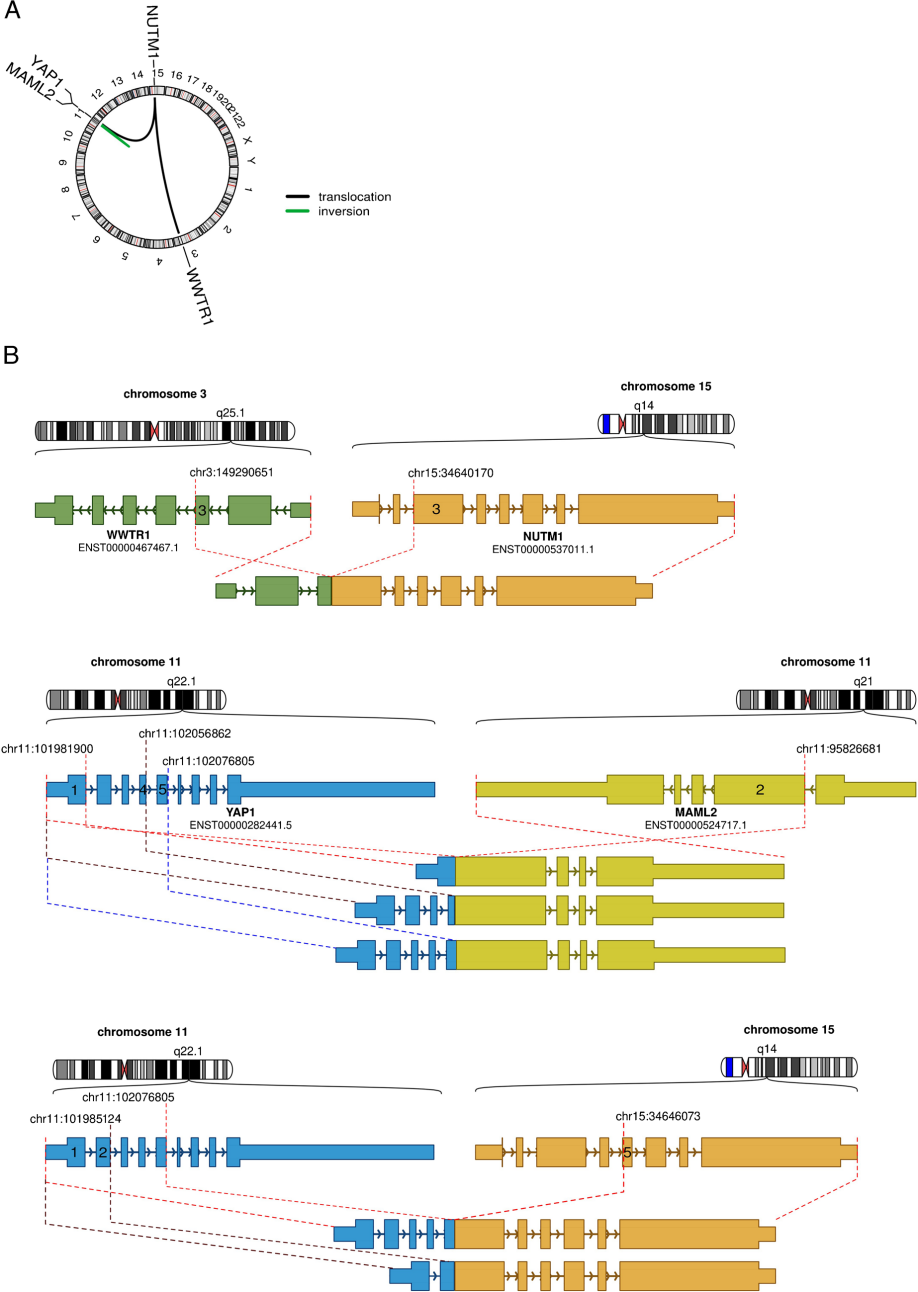
Detected gene fusions in skin-analogue poroid neoplasms. **A** Circos plot depicting genomic localization of gene fusion partners and the nature of the rearrangement. **B** Visualization of individual gene fusions. Shown are the exon (boxes)/intron (lines and arrows) compositions of individual genes and the resulting fusion constructs. Exons at breakpoints are indicated by their exon number and their respective genomic position

Targeted RNA sequencing revealed a *YAP1* fusion in nine cases (fused to *MAML2* in four and to *NUTM1* in five cases) and a *WWTR1::NUTM1* fusion in one case. All fusions were in frame (Fig. 6).

## Discussion

Cutaneous porocarcinoma is rare with a prevalence in unselected skin biopsies of 0.004% [16]. The disease affects both sexes equally with a mean age of 67 years [17]. The head and neck region is the most common site followed by the extremities [17, 18]. Due to its rarity and the immunohistochemical overlap with the non-keratinizing cutaneous squamous cell carcinoma (SCC), porocarcinoma is probably still underrecognized by general surgical pathologists. However, its appropriate recognition and separation from conventional SCC is mandatory as porocarcinoma has a higher tendency for systemic metastases. In one large metaanalysis study, the metastatic rate was 31% with 57% of metastatic cases showing regional lymph node disease [17].

Histologically, porocarcinoma displays a variable admixture of monomorphic smaller basaloid-looking poroid epithelial cells admixed with variable component of larger eosinophilic cuticular cells [9, 10]. A variable ductal component may be present, but this is usually a focal finding, and many tumors lack overt ductal differentiation [9, 10, 16]. At immunohistochemical level, the neoplastic cells of porocarcinoma are uniformly positive with the squamous cell markers (pankeratin, high molecular weight keratins and p63/p40). In addition, EMA and CEA may be valuable to highlight subtle ductal differentiation and may show diffuse expression in the neoplastic cells [16]. Rarely, variant histology (clear cell change, frankly squamous features, sarcomatoid pattern, sebaceous differentiation) may be observed, complicating the differential diagnostic consideration [9, 10, 16]. Cutaneous porocarcinoma may develop either de novo or from preexistent benign poroma. Distinction is mainly based on frankly malignant cytology and unequivocal invasive growth, but the spectrum seems continuous with occasional cases spanning the border between the two ends of the disease.

To our knowledge, only a single case of poroid carcinoma originating in the parotid gland of a young adult has been reported as such before [11]. Two other cases originating in the parotid and the nasopharynx have been reported as non-keratinizing SCC [12, 13]. However, a new case of poroid squamous cell carcinoma/squamoid porocarcinoma (originating in the parotid gland of a 62-year-old female) was published ahead-of-print during preparation of this revision [19]. In the current study, we reviewed our previously reported cases and added seven unpublished cases to delineate the clinicopathological and molecular profiles of poroid neoplasms originating in extracutaneous head and neck sites.

Skin-analogue poroid neoplasms of the head and neck seem to recapitulate the demographic, morphological and molecular spectrum of their cutaneous counterparts. Notably, they affect predominantly middle-aged adults at a mean age of 49 years but may occur in the very young, similar to their cutaneous counterparts. Their histology varies from relatively bland looking tumors indistinguishable from cutaneous eccrine poroma with frequent prominent cystic features (dermal duct tumor-like pattern) to tumor with frankly high-grade features associated with extensive geographic necrosis and brisk mitotic activity. The major salivary glands (mainly the parotid) are the main site of origin followed by other minor salivary gland sites.

The histogenesis of poroid neoplasms at these extracutaneous sites is unclear. We have not observed distinct preexisting benign poroma or pleomorphic adenoma component in the high-grade cases, indicating that these high-grade tumors likely developed de novo. However, prominent cystic features in the bland-looking cases and connection to the duct system (observed altogether in four cases) suggest origin from the excretory duct segment of the salivary glands. Likewise, the cystadenoma-like case is another argument for ductal origin, but this remains currently speculative. One reported case originated presumably from a lymphadenoma, but the frequently observed occurrence of prominent lymphoid reaction and cystic features in our bland-looking cases argues for a lymphadenoma-like pattern rather than genuine preexistent lymphadenoma. Although the salivary glands do not possess a terminal sweat duct analogue, origin of skin adnexal-type neoplasms in the salivary glands and of salivary-type neoplasms in the skin has been increasingly recognized, indicating that many if not most entities may on occasion develop in these anatomically and embryologically distinct organs. Notably, tumors at these two distinct sites share not only morphological but also genotypic features [20, 21], albeit with significantly varying frequencies and some site-dependent inherent morphological and biological properties. All of our cases have shown gene fusion characteristic of their cutaneous poroid counterparts [22]. *YAP1::MAML2* and *YAP1::NUTM1* fusions have been detected in 63.6% of cutaneous porocarcinomas [22]. The *WWTR1::NUTM1* fusion variant is much rare and has been reported to date in only two cases of poroma [22, 23]. A novel *YAP1::MAML3* fusion has been reported in a case of spindle cell porocarcinoma [24]. However, the genetic landscape of cutaneous porocarcinoma has been expanding recently to include *PAK1/2/3* fusions [25, 26]. In the current study, we detected similar genetic findings with *YAP1::MAML2* and *YAP1::NUTM1* fusions being the most prevalent fusion variant (detected with similar frequency in four and five of nine cases, respectively), while the rare *WWTR1::NUTM1* fusion was detected in one case.

Recently, surrogate immunohistochemistry has emerged as a powerful tool to screen for and/or identify fusions in poroid neoplasms [27, 28]. Notably, loss of YAP1 c-terminus antibody combined with NUTM1 antibody expression can largely and reliably predict the underlying *YAP1::NUTM1* fusion in poroid skin neoplasms [27, 28]. On the other hand, loss of YAP1 with negative NUTM1 IHC points to a probable *YAP1::MAML2* or rare related fusions [27, 28]. Tumors with *WWTR1::NUTM1* fusion are expected to display NUTM1 expression with preserved YAP1. It is, however, noteworthy that Rb1 knockdown was shown to repress YAP1 protein expression in experimental studies [29]. This explains the frequent loss of YAP1 in Rb1-deficient skin cancer lacking *YAP1* fusions and underlines the need to include Rb1 immunohistochemistry into the surrogate immunopanel (Table 3). Accordingly, tumors with loss of both YAP1 and Rb1 need molecular testing to prove or rule out an underlying *YAP1* fusion.

**Table 3:** Surrogate immunohistochemistry to predict gene fusions in poroid neoplasms

YAP c-terminus IHC	NUTM1 IHC	Rb1 IHC	Predicted fusion type
Loss	Neg	Retained	*YAP1::MAML2*
Loss	Neg	Loss	Either no *YAP1::MAML2* fusion, or *YAP1::MAML2* fusion with secondary Rb1 loss; molecular testing mandatory
Loss	+	Retained	*YAP1::NUTM1*
Loss	+	Loss	Either *YAP1::NUTM1* with secondary Rb1 loss or *non-YAP1::NUTM1* fusion
Retained	+	Not relevant	*non-YAP1::NUTM1* fusion, possibility of: - *WWTR1::NUTM1 (poroid neoplasm)* - BRD3/4/NSD3::NUTM1 (NUT carcinoma*)

*These genotypes define conventional NUT carcinomas and are not encountered in poroid carcinomas

Regarding the differential diagnosis of skin-analogue salivary poroid carcinoma, non-keratinizing SCC is the major consideration, given their shared squamous immunophenotype [1]. Primary SCC of the major salivary gland has been elusive and most represent metastatic SCC of skin origin [1]. Histologically, poroid carcinoma is usually monotonous and lacks the significant polymorphism and bizarre cytology of metastatic SCC. They also grow in multiple lobules and solid nests and are not dyscohesive as in many metastatic SCC. Tumors with clear cell morphology need to be differentiated from clear cell myoepithelial carcinoma, clear cell variant of mucoepidermoid carcinoma, and clear cell carcinoma [3, 30].

More critical is the distinction between NUTM1-positive poroid carcinoma and conventional NUT carcinoma. Currently, NUT carcinoma is not considered a “midline” disease anymore. With the widely increasing use of screening NUT immunohistochemistry, this highly aggressive entity has been documented at nearly any organ/sites including lateralized organs. NUT carcinoma may present rarely as primary salivary gland malignancy and poses great diagnostic challenge as it closely mimics primary or metastatic SCC and porocarcinoma histologically [7]. On the other hand, distinguishing NUT carcinoma from *NUTM1*-rerranged poroid carcinoma is challenging [31]. NUT carcinomas of the salivary glands recapitulate their counterparts from other sites and may display purely basaloid, abruptly keratinizing squamous or anaplastic morphology [7]. They express frequently squamous cell markers, similar to porocarcinoma. However, careful evaluation of cytoarchitectural features is valuable in the initial screening for potential poroid carcinoma. At the molecular level, NUT carcinomas are characterized by *BRD4::NUTM1* fusions in majority of cases followed (with almost similar frequency) by *BRD3::NUTM1*, *NSD3::NUTM1*, and *ZNF::NUTM1* fusion variants [32]. The shared anatomic site, squamous phenotype, and expression of NUTM1 indicate the necessity of molecular profiling to reliably separate NUT carcinoma from *NUTM1*-rerranged poroid carcinoma at head and neck sites.

*MAML2*-rearranged poroid carcinomas are at high risk to be confused with mucoepidermoid carcinomas that show variant (non-mucinous) or high-grade histology, as both display a squamoid morphology and immunophenotype and share MAML2 rearrangements. A more diffuse p63/p40 should suggest a poroid neoplasm, but might be rarely seen also in high-grade mucoepidermoid carcinoma. Loss of the YAP1 C-terminus protein is highly valuable surrogate for *YAP1::MAML2* fusion. In challenging cases, definitive molecular diagnosis should be based on either *CRTC1/3::MAML2* fusion transcript detected by RT-PCR or NGS, but not on *MAML2* FISH. Finally, skin analogue poroid carcinoma in the head and neck should be distinguished from salivary duct carcinoma and carcinoma ex pleomorphic adenoma with prominent basal-like/squamous differentiation. At the end, thorough sampling and molecular profiling represent the mainstay to recognize extra-cutaneous poroid carcinomas and separate them from their many morphophenotypical mimics that may frequently, occasionally, or rarely present at these sites. Last but not least, cutaneous porocarcinoma may metastasize to salivary glands or other head and neck sites and should be ruled out by clinical evaluation and exclusion of a skin primary.

In the appropriate clinical and morphological context, the detected *YAP1/WWTR1::MAML2/NUTM1* fusions are highly specific for poroid neoplasms. Notably, among > 1000 head and neck cases tested at our centers by targeted RNA sequencing, similar fusions have not been encountered in any other entities, underlining the relative specificity of these fusions for poroid neoplasms (own unpublished data). However, *YAP1*::*MAML2* fusions have been reported in rare cases of diverse malignancies including nasopharyngeal carcinomas, SCC of the tongue, and other non-head and neck entities [33, 34]. The relationship of some of these rare reports on *YAP1::MAML2*-fused head and neck carcinomas to poroid carcinoma remains unclear, as they might have represented unrecognized poroid carcinomas.

In summary, we reported 10 morphologically and molecularly verified poroid neoplasms occurring at extracutaneous head and neck sites (salivary glands, nasopharynx, and the mandible). Our study introduces “skin-analogue poroid neoplasms” as a novel family of tumors in the head and neck, which mirror their cutaneous counterparts clinically, morphologically, biologically, and molecularly. These tumors are probably underrecognized at these unreported/unexpected anatomic sites. Hence, including poroid neoplasms into the differential consideration of carcinomas showing squamous phenotype in the head and neck should positively enhance detection and better characterization of these rare aggressive carcinomas. Their dichotomous division into benign poroid tumors and poroid carcinomas remains to be further characterized.

## Data Availability

The datasets generated during and/or analyzed during the current study are not publicly available, but are available from the corresponding author on reasonable request.
